# Investigation of a Perspective Urban Tree Species, *Ginkgo biloba* L., by Scientific Analysis of Historical Old Specimens

**DOI:** 10.3390/plants13111470

**Published:** 2024-05-26

**Authors:** Szilvia Kisvarga, Dóra Hamar-Farkas, Katalin Horotán, Csaba Gyuricza, Katarína Ražná, Matúš Kučka, Ľubomír Harenčár, András Neményi, Csaba Lantos, János Pauk, Ádám Solti, Edina Simon, Dina Bibi, Semonti Mukherjee, Katalin Török, Andrea Tilly-Mándy, László Papp, László Orlóci

**Affiliations:** 1 Ornamental Plant and Green System Management Research Group, Institute of Landscape Architecture, Urban Planning and Garden Art, Hungarian University of Agriculture and Life Sciences (MATE), 1223 Budapest, Hungary; kisvarga.szilvia@uni-mate.hu (S.K.); nemenyi.andras.bela@uni-mate.hu (A.N.); orloci.laszlo@uni-mate.hu (L.O.); 2 Department of Floriculture and Dendrology, Institute of Landscape Architecture, Urban Planning and Garden Art, Hungarian University of Agriculture and Life Sciences (MATE), 1223 Budapest, Hungary; tillyne.mandy.andrea@uni-mate.hu; 3 Institute of Biology, Eszterházy Károly Catholic University, 3300 Eger, Hungary; horotan.katalin@uni-eszterhazy.hu; 4 Institute of Agronomy, Hungarian University of Agriculture and Life Sciences (MATE), 1118 Gödöllő, Hungary; 5 Institute of Plant and Environmental Sciences, Faculty of Agrobiology and Food Resources, Slovak University of Agriculture in Nitra, 94976 Nitra, Slovakia; katarina.razna@uniag.sk (K.R.); xkucka@uniag.sk (M.K.); xharencar@uniag.sk (Ľ.H.); 6 Cereal Research Non-Profit Company, 6726 Szeged, Hungary; csaba.lantos@gabonakutato.hu (C.L.); janos.pauk@gabonakutato.hu (J.P.); 7 Department of Plant Physiology and Molecular Plant Biology, Eötvös Loránd University, 1117 Budapest, Hungary; adam.solti@ttk.elte.hu; 8 Eötvös Loránd Research Network, University of Debrecen, 4032 Debrecen, Hungary; simon.edina@science.unideb.hu; 9 Anthropocene Ecology Research Group, Department of Ecology, University of Debrecen, 4032 Debrecen, Hungary; dinaangel172@gmail.com (D.B.); semontimukherjee@gmail.com (S.M.); 10 Eotvos Lorand Res Network (ELKH), Institute of Plant Biology, Biological Research Centre, 6722 Szeged, Hungary; torok.karolyne@brc.hu; 11 Füvészkert Botanical Garden, Eötvös Loránd University, 1053 Budapest, Hungary; papp.laszlo@fuveszkert.elte.hu

**Keywords:** *Ginkgo biloba*, plant genetics, micromorphology, historical tree, stress tolerance, climate change

## Abstract

In this study, we examined over 200-year-old *Ginkgo biloba* L. specimens under different environmental conditions. The overall aim was to explore which factors influence their vitality and general fitness in urban environments and thus their ability to tolerate stressful habitats. In order to determine this, we used a number of different methods, including histological examinations (stomatal density and size) and physiological measurements (peroxidase enzyme activity), as well as assessing the air pollution tolerance index (APTI). The investigation of the genetic relationships between individuals was performed using flow cytometry and miRNA marker methods. The genetic tests revealed that all individuals are diploid, whereas the lus-miR168 and lus-miR408 markers indicated a kinship relation between them. These results show that the effect of different habitat characteristics can be detected through morphological and physiological responses, thus indicating relatively higher stress values for all studied individuals. A significant correlation can be found between the level of adaptability and the relatedness of the examined individuals. These results suggest that *Ginkgo biloba* L. is well adapted to an environment with increased stress factors and therefore suitable for use in urban areas.

## 1. Introduction

### 1.1. Origin of Ginkgo biloba (*L*.) and Its Emergence in the European Carpathian Basin

The origin of wild *Ginkgo biloba* populations in China has long been disputed. The extant *Ginkgo biloba* populations in the Dalou Mountains of Southeast China most likely represent populations that were not affected by human activities [1], but Zhao [2] and Xiang et al. [3] argue that the Tianmu Mountains and the Dahong Mountains are also natural habitats of the taxon. The very first specimens arrived in European countries as cuttings [4]. The first horticultural record on existing *Ginkgo biloba* specimens in the present-day territory of Hungary dates back to 1808 when trees were already cultivated along with pine species in arboretums [5], but later on the taxon spread in dendrological collections rapidly in the 19th century. The very first specimens of *Ginkgo biloba* were planted in the garden which used to belong to Count Antal Festetics back in the early 1800s; the area was gradually enclosed by Budapest and is currently known as the Botanical Garden of Eötvös Loránd University. [6]. In the palace garden of the Festetics counts in Keszthely town, Hungary, another specimen was planted under the ownership of count György Festetics [5,7]. These approximately 200-year-old specimens have grown to mature trees over the years and represent a major botanical, historical, and touristic value. However, the exact origin of the propagating materials of these trees remains uncertain. These old trees not only survived the storms of history, but also extremities in the environmental conditions in the past two centuries; thus, they represent specimens of outstanding genetic potential.

### 1.2. The Unique Feature of Ginkgo biloba (*L*.)

Among the world’s estimated 100 000 tree species, one of the best known and most easily recognized is the gymnosperm *Ginkgo biloba* (L.; *Ginkgophyta*) which stands out for its unique properties, amazing history, and long association with humans [4]. *Ginkgo biloba* is a woody species of the monotypic *Ginkgoaceae* and *Ginkgoales* [5,6,7,8,9,10]. Although it is the only to-date surviving member of the once diverse and dominant family and order, in the last centuries, it became widely planted as an ornamental, food, and medicinal plant [5].

One of the defining characteristics of *Ginkgo biloba* is that it is dioecious, but it is also capable of sexual switching, and this–among other characteristics–allows it to have a higher tolerance towards extreme conditions, such as a change in climate or habitat conditions [11]. *Ginkgo biloba* is one of the most widely used street trees in settlements in temperate areas of the world [12]. It has been successfully cultivated in urban areas with high air pollution, poor drainage, compacted soil, and drought [13].

### 1.3. Ploidity and Genetic Background of Ginkgo biloba (*L*.)

*Ginkgo biloba* can be characterized to have a somatic diploid chromosome number of 2n = 24 [14]. The draft genome of *Ginkgo biloba* was compiled with a size of 10.6 G bp, 76.6% of which was considered as repetitive sequences [15]. The renewed analysis of the genome indicated, indeed, a 9.9 G bp length of the genome [16]. Both polyploid and haploid samples have been recorded among cultivated specimens. Since among specimens of wild populations, no polyploid samples were collected [17], polyploidy should be considered a trait that arose under horticultural selection. In the last 20–30 years, the increased interest in *Ginkgo biloba* as an ornamental tree and thus the selection of plants with attractive foliage and growth form contributed to the propagation of polyploid lines [18,19]. Wild populations overall show a narrow genetic variation, e.g., in a form of reduced AFLP polymorphism that approves the *Pleistocene refugia* hypothesis [20]. Lhcb genes are expressed in a highly light-dependent manner [21], which is not seen in pinophytes but rather is characteristic of angiosperms and thereby exhibits a characteristic of this group that goes beyond its appearance. In addition, it is worth mentioning that the chloroplast genome of *Ginkgo biloba* also contains an inverted repeat encoding a 17 k bp ribosomal RNA [22].

### 1.4. Foliar Macromorphology and Histology

The macromorphological characteristics of *Ginkgo biloba* have been preserved in a wide range of fossil remains, and thus its change can be traced throughout the history of the Earth. In a similar way, recent *Ginkgo biloba* specimens also show a uniform macromorphology and notable genetic stability [23]. Regarding the foliar macromorphology, the fan-shaped lamina, thick cuticle, and glutinous leaf surface are characteristic and make the leaves resistant to decay upon abscission [12]. Though foliar micromorphology in consent, micromorphology traits such as stomatal distribution are rather a matter of alterations by environmental and genetic backgrounds [24,25]. Stomatal conductance fundamentally affects the transpiration water loss of leaves and thus the water status of the shoot tissues. *Ginkgo biloba* leaves are classified as hypostomatoside [26]; despite this, stomata have occasionally also been observed on the adaxial surface [27]. The type of stomata is haplochelic, and their location on the leaf is distributed unevenly between the leaf veins. The average length of the stoma guard cells is 45.9–55.7 μm. The stroma guard cells have a mean length of between 45.9 and 55.7 μm [19]. The density of the stomata varies from 100 to 140 mm^−2^ [28], and they are sensitive to various environmental factors, including humidity, irradiance, and atmospheric CO_2_ [29].

Since stomata density is a primary trait that determines water use efficiency, it is highly affected by various environmental stresses such as salt stress [24]. Nevertheless, stomatal density is also determined by genetic traits, as it was shown on *Paspalum rawitscheri* [30], *Aleurites montana* [31], and *Sorghum bicolor* [32] lines. In *Arabidopsis thaliana*, the average stomatal density and stomatal size were shown to correlate negatively [33]. In *Trapa natans* var. *bispinosa*, variations in the stomatal density were shown to correlate to genetic variability [34]. In *Ginkgo biloba*, the stomatal density was also linked to genetic traits. Flow cytometry analysis on European *Ginkgo biloba* collections (>2200 individuals, approx. 200 varieties) indicated a correlation between ploidy differences and stomatal density [19].

The aim of the study was therefore to assess the urban suitability of *Ginkgo biloba* in the Carpathian Basin. This was assessed using old, historical trees that are more than 200 years old and face a number of environmental challenges. The three selected species are found in different environmental conditions—in crowded cities (Budapest), small towns (Keszthely), and villages (Acsád). Based on this, our aim was to assess the condition of the three trees by performing histological and stress tests, as well as an assessment of urban resilience. An important objective of the study was also to assess whether there is a genetic link between the three specimens studied, which would allow for a more comprehensive evaluation of the old trees and a better conservation of the plant genetic and historical heritage, thus allowing for comparison with other *Ginkgo biloba* specimens from the Carpathian Basin and further afield.

## 2. Results

A series of measurements were made on the *Ginkgo biloba* specimens studied in order to detect the response of the plants to abiotic stress in the urban environment, as well as to investigate whether this induced any histological alteration in the leaf (stomatal density, size).

### 2.1. Micromorphology

**Figure 1:**
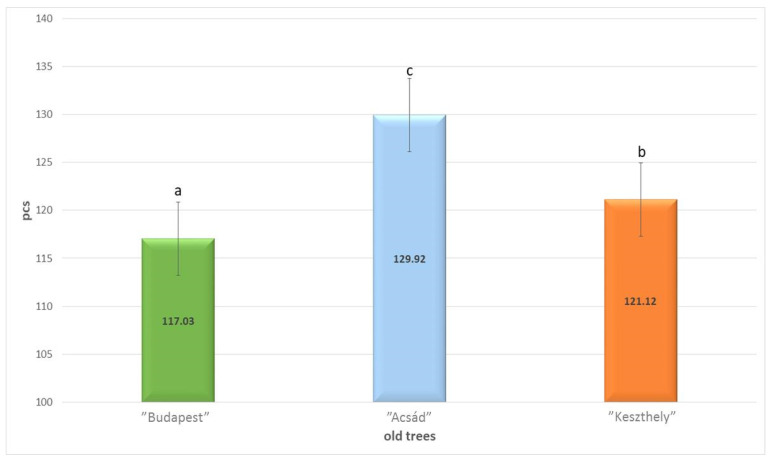
Average stomata number of the three old *Ginkgo biloba* trees named by their locations (Games–Howell, *p* < 0.05). Different letters (a–c) indicate significantly different groups. ANOVA: *p* = 0.000. (pcs—pieces).

Examining the leaf data from different sections of the three historical *Gingko biloba* trees, it was found that the average numbers of stomata “Acsád” tree (129.92 (c) pcs) were significantly higher (*p* = 0.000) those of the “Budapest” tree (117.03 (a) pcs) and “Keszthely” trees (121.12 (b) pcs) (Figure 1).

**Figure 2:**
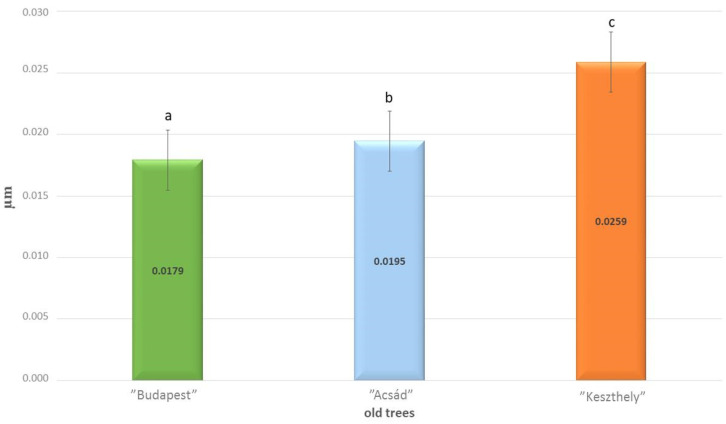
Average stomata size of the three old *Ginkgo biloba* specimens (Games–Howell, *p* < 0.05). Different letters (a–c) indicate significantly different groups. ANOVA: *p* = 0.000.

For the three studied individuals, the stomatal length (Figure 2) is not correlated with the stomatal number results (Figure 1), which is contradictory to the literature references.

The shortest stomatal length was measured in the “Budapest” tree (0.0179 (a) µm), while the longest was found in the “Keszthely” tree (0.0259 (c) µm). There is a significant difference (*p* = 0.000) between the measured data.

### 2.2. Air Pollution Tolerance Index (APTI)

The data obtained from the APTI studies (Table 1) were evaluated on two specific scales [35,36], for which the individuals tested can be classified in the same category (intermediate and sensitive). Looking at the measured values, there was no major difference between the “Acsád” and “Keszthely” samples concerning moisture (Relative Water Content, RWC), ascorbic acid (AAC), chlorophyll, and pH values. This might be explained by the fact that the two trees have almost identical habitat characteristics in the urban environment. In the case of the “Budapest” tree, the APTI values point to the fact that it is considered to be in the ‘sensitive’ category according to both scales.

### 2.3. Determination of Ploidy Level in Gingko biloba Specimens

**Figure 3:**
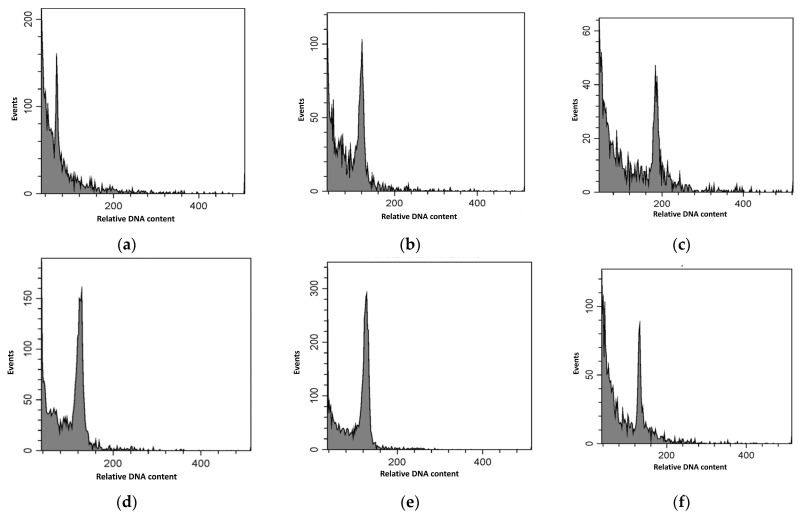
Flow cytometric analyses of *Ginkgo biloba* L. samples: histograms demonstrate the relative DNA content of (**a**) “Chris Dwarf”, haploid control; (**b**) seed-derived *Gingko biloba* L., diploid control; (**c**) “Knapek’s triploid”, triploid control; (**d**) “Acsád”; (**e**) “Budapest”; and (**f**) “Keszthely” samples.

The level of ploidy in *Ginkgo biloba* L. was determined by flow cytometric analysis. The measurements revealed the relative DNA content of the tested samples in comparison with the haploid, diploid, and triploid controls. The ploidy level of the “Acsád”, “Budapest”, and “Keszthely” samples was determined as diploid based on the histogram of flow cytometric analyses (Figure 3).

#### 2.3.1. Genetic Evaluation Based on miRNA Markers

In total, five different miRNA-based markers were used in the diversity analysis of the *Ginkgo biloba* samples of various origin. Markers lus-miR168 and lus-miR408 are representatives of stress-sensitive markers [37, 38,39]. The crossgenera transferability potential of miRNA-based markers has been confirmed by several studies [40,41].

**Figure 4:**
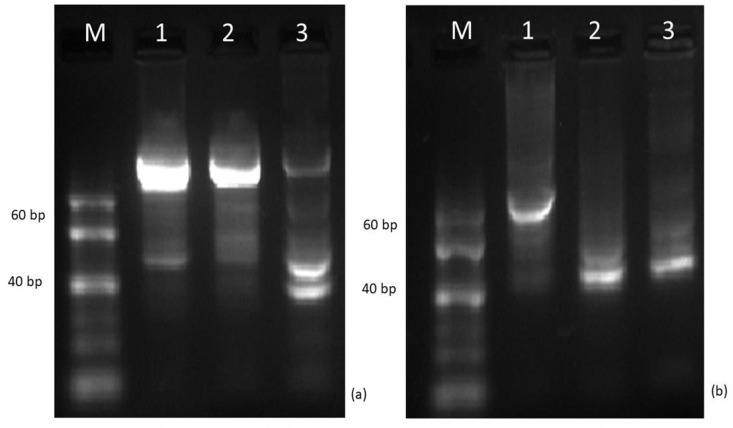
Order of samples on the electrophoretograms lus-miR168 (**a**) and lus-miR408 (**b**); 1—“Acsád”; 2—“Keszthely”; 3—“Budapest” (photo: Ražná, Slovakia).

The presence of an amplified PCR product of the expected size 80 bp has been confirmed in the genome of analyzed samples (Figure 4).

Both types of stress-sensitive markers lus-miR168 and lus-miR408 pointed out the genome specificity of samples originated from environmentally less polluted areas (“Acsád” or “Keszthely”).

#### 2.3.2. Determination of Stress Enzyme Activity

**Figure 5:**
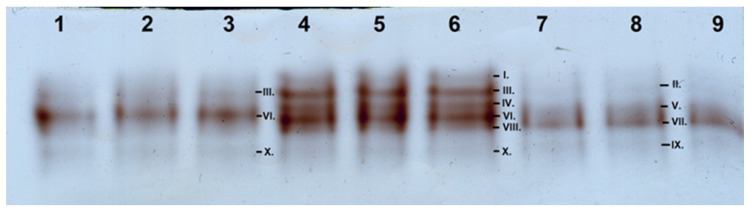
Separation of the isoforms of class III peroxidases in *Ginkgo biloba* samples: 1–3: “Acsád”; 4–6: “Budapest”; and 7–9: “Keszthely”.

**Figure 6:**
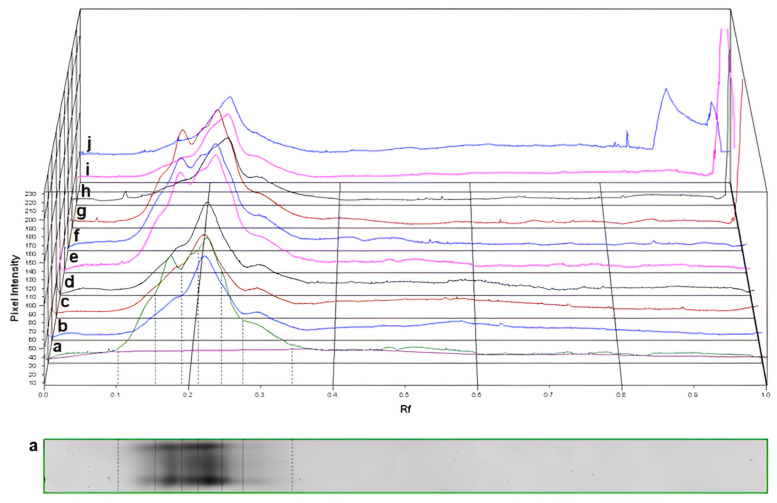
Unnormalized densitograms of class III activity-stained native polyacrylamide gel electrophoretograms of *Ginkgo biloba* samples: a: reference sample “Budapest”; b–d: “Acsád”; e–g: “Budapest”; h–j: “Keszthely”.

The separation of the POD isoforms by native PAGE resulted for all the three plant specimens in a total of 10 isoforms, which were identified as R_f,I_: 0.147; R_f,II_: 0.162; R_f,III_: 0.174; R_f,IV_: 0.201; R_f,V_: 0.211; R_f,VI_: 0.223; R_f,VII_: 0.233; R_f,VIII_: 0.247; R_f,IX_: 0.276; and R_f,X_: 0.291 (Figure 5 and Figure 6).

**Figure 7:**
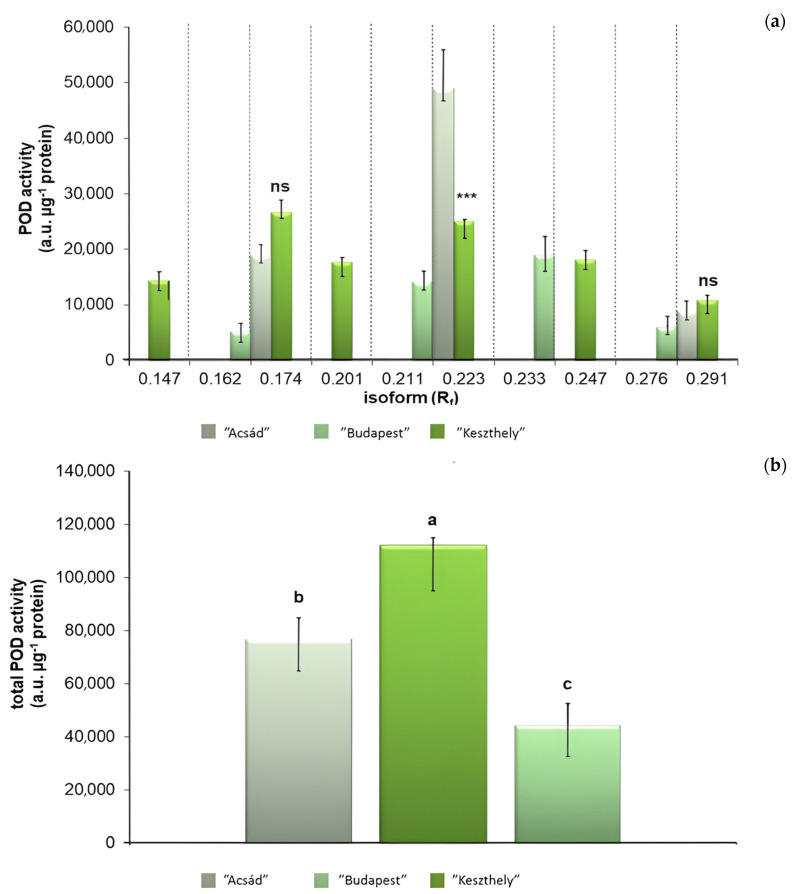
Class III peroxidase (POD) activity of the (**a**) separated isoforms, indicated by retention factors (R_f_) and the total POD activity (**b**) in ‘“Acsád” (left), “Budapest” (middle), and “Keszthely” (right) samples. To compare differences, Student’s *t* test (**a**) and one-way ANOVA with Tukey–Kramer post hoc test (**b**) were performed (*p* < 0.05). On (**a**), asterisks indicate significant difference, and ns indicate not significant. On (**b**), letters indicate statistical groups.

None of the plant samples showed the activity of all the isoforms: the highest number (six) of isoforms were detected in the “Budapest” specimen. Although the activity of only three isoforms was detected in the “Acsád” individual, all these were also present in the “Budapest” individual. In the “Keszthely” sample, the activity of four POD isoforms was detected. However, all these isoforms proved to be distinct from those in the “Acsád”and the “Budapest” individual. Differences in the total POD activity among the three specimens were significant. Nevertheless, statistical differences between the activities of R_f,III_: 0.174 and R_f,X_: 0.291 when comparing the “Budapest” and the “Acsád” individuals were not significant (Figure 7).

## 3. Discussion

To understand how old tree specimens survived a long time, withstanding the environmental challenges, case studies could provide a good help. Here, we analyzed three of the oldest *Ginkgo biloba* specimens that can be found in a relatively small geographical area. Although early *Ginkgo biloba* plantings took place in European dendrological collections, mansion gardens and arboretums were supposed to originate from a restricted genetic source, and a relationship between the investigated old specimens has remained unknown. In our experiments, we sought to find out the genetic traits and stress tolerance of the three oldest *Ginkgo biloba* species in the Carpathian Basin, but they are estimated to be over 200 years old. Their historical landscape evaluation is in an ongoing process; however, with these horticultural and biological measurements, it can be complemented and continued in the future. Beyond these, establishing a possible genetic relationship was also an important part of our measurements.

### 3.1. Health and Physiological Status of the Old Trees

We received results on the health and physiological status of the trees. In this way, we arrived at an idea of the condition of the individuals living in Carpathian Basin for almost 200 years and their genetic characteristics. Thus, we aimed to answer why old *Ginkgo biloba* specimens survived in the Carpathian Basin, despite the supposed climate extremities, and how they are related to each other.

**Figure 8:**
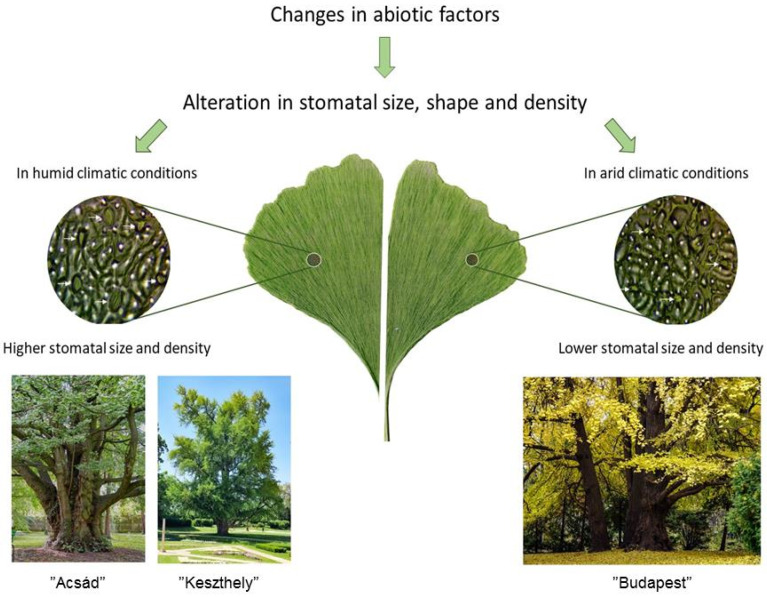
Stomal size and density in the light of abiotic influences (Horotán, 2024).

The climate of the Acsád village is primarily affected by the close distance from the Alps; as a result, the maximum temperatures are lower than areas dominated by continental climate influences in the Carpathian Basin. Keszthely town is located near Lake Balaton, which is the largest freshwater lake in Europe. Since the “Keszthely” individual is located in a mansion park of a small town, attracting a large number of tourists, urban effects are supposed to be more significant in comparison with those affecting the “Acsád” specimen. Since the “Budapest” specimen is located in the Botanical Garden of the Eötvös Loránd University, which is nowadays close to the center of the city of Budapest—a densely populated area—the microenvironment is affected by constant heavy traffic and air pollution, as well as other urban effects such as heat emission. Although the urban effect is reflected in the health of the tree, this result also correlates with the Razna et al. [11] results on urban stress tolerance, thus indicating that the species is highly resilient to extreme environmental factors. The stomatal number and length values could partially indicate the genetic diversity of the trees, since their measurements are not inversely proportional to each other, as the previously mentioned literature suggests (Figure 8).

The results of Kiani-Pouya et al. [24] correlate with measurements showing that stomatal size is strongly influenced by external stress effects. Comparing our data with those of Drake et al. [42], we assume that leaves modified for greater gas exchange have smaller stomata and faster dynamic characteristics. This can be explained by the fact that the higher water potential of the larger stomata requires more energy from the stoma, but the faster response time of the smaller stomata can help offset this. Most studies of stomata have focused on their development and responses to environmental stressors [43]. Smarda et al. have shown that stomatal density and attributes can be determined by genetic traits in this species [19], with which our results are also consistent.

### 3.2. Origin of the Ginkgo biloba Individuals

We performed a micromorphological, physiological, and genetic comparison of the three oldest *Ginkgo biloba* individuals in Hungary in the Carpathian Basin. The purpose of our experiment is to determine whether there is a genetic relationship between the breeds. We based our assumption on the fact that the first *Ginkgo biloba* individuals came to the European continent in the 18th century, thus undergoing urban stress tolerance, as discussed by Fekete et al. [5] and Papp [6] in their work.

Stomata appeared more than 410 million years ago and are absent in some taxa of the earliest land plants in evolution, but they are present in all other land plants [44]. However, the development of stomata has been affected both by genetic and environmental factors. Sapra [45] showed in triticale (×*Triticosecale*) that, together with the ploidy increase, the number of stomata decreased, but the size of the stomata increased. We suggest that the ploidy level of examined *Ginkgo* trees can be different. Šmarda et al. [19] found that in *Ginkgo biloba*, the ploidy level even within an individual may vary, thus we cannot exclude the possibility that this phenomenon might have affected our samples too. Therefore, we cannot confirm that all three individuals are diploid. To complement the miRNA analysis, data of the comparison of the stomata number indicate that the higher stomata number of the “Acsád” tree may reflect a genetic property in other than the two other specimens. Genotypic changes in stomatal density and size may also be decisive [46]. Also, the profile of lus-miR168-based loci has pointed out on the genomic similarity of the *Ginkgo* tree from the Budapest and Keszthely cities, while the tree from Acsád belongs to a different genetic group. In this context, the stomata number data are consistent with this result. By comparing the results of the stoma size with the loci profile of the lus-miR408 marker, we found that the individuals from Keszthely and Acsád may belong to a separate group. The lus-miR168 and lus-miR408 markers exactly support this result. In addition to all this, it can be concluded that genetic tests can be used to investigate the genetic comparison of the *Ginkgo biloba* trees.

### 3.3. Urban Stress Tolerance of Old the Ginkgo biloba Trees

However, the analysis of POD isoform distribution indicate a closer connection between the “Acsád” and the “Budapest” specimens, whereas the “Keszthely” specimen appears to be distinct. Since the induction of POD isoforms may also vary in an environmental stress-dependent manner [47], we suppose that the result was rather affected by climatic effects and not by genetic traits.

Compared to previous assumptions [7,48], our results show that considering the weather conditions, the Budapest specimen is exposed to the greatest level of stress. The findings of the APTI series indicate that the urban stress tolerance of the “Keszthely” and “Acsád” individuals was found to be average, and there was no statistically significant difference measured between them, but the “Budapest” individual was found to be more sensitive to environmental stress. The higher the level of stress, the higher the POD activity level and thus the better the current state of the plant can be determined. As the POD activity was highest in the leaves of the Budapest specimen, the results also support the hypothesis that the urban environment resulted in significantly higher stress levels for the trees. Therefore, the POD can be considered as an indicator of stress levels rather than as a biomarker of the individual. The miRNA-based analysis shows genetic variation.

## 4. Materials and Methods

### 4.1. Investigated Specimens and Their Locations

This study was focused on three historical *Ginkgo biloba* L. specimens that are among the first of their species to have been introduced and planted in Europe and are the oldest known specimens in the Carpathian Basin. The investigated trees are found in parks. These parks are parts of settlements of different sizes and populations, which have different levels of air pollution due to their size. The three sites can be characterized with similar temperate continental climatic conditions.

The “Acsád” specimen is located in the Acsád mansion park, Acsád, Hungary. According to historical records, the tree, currently 18 m tall, was planted in 1808. The tree might have been damaged; the trunk has branched low. Although the majority of the flowers are gynoecious, a minor fraction of flowers are always androecious. Trunk circumference is 527 cm.

The “Keszthely” specimen is located in the Keszthely mansion park, Keszthely, Hungary. The height is currently 23 m. Trunk circumference is 412 cm. Flowers are gynoecious. The tree was a seedling, probably 2–5 years old when it was planted [49].

The “Budapest” specimen is located in the present-day Botanical Garden of the Eötvös Loránd University, Budapest, Hungary. The specimen is generally considered to be of over 200 years of age and planted around 1801. The height of the specimen exceeds 30 m. Flowers are androecious. The specimen is surrounded by two more *Ginkgo biloba* trees, which are almost the same age. Trunk circumference is 382 cm.

All examinations were carried out in 2022. Leaf samples were collected on 6 July 2022. All three specimens are located in the Carpathian Basin at similar altitude.

### 4.2. Density and Size of Stomata

Stomatal density was measured using leaf surface replicates. Leaf surfaces were coated with colorless varnish at 3 well-separated points on the abaxial side, and after 15 min of drying, replicates were removed.

**Figure 9:**
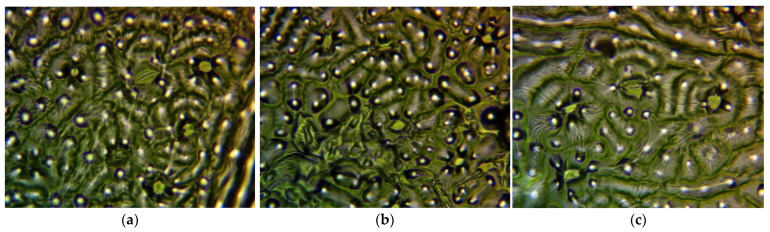
Selected micrographs of the replicates applied in stomatal density and size calculations. (**a**) “Budapest”; (**b**) “Acsád”; (**c**) “Keszthely” sample.

Using a Euromex bScope BS.1153-PLi type microscope (mounted with PLi 4/0.1 lens at 40× magnification, Euromex Microscopen B.V., Arnhem, The Netherlands) with WF120×/20 eyepiece and Levenhuk m1400 plus camera (Levenhuk, Inc., Budapest, Magyarország), on each of the replicates, a 5 mm × 5 mm area was analyzed (Figure 9).

Since no or very limited number of stomata appeared on areas laying over the leaf veins, these data were rejected from the analysis. Altogether, replicates were prepared in 3 repetitions per leaf, and 15 leaves per individual were subjected to the analysis. Stomatal density was determined using 5 repetitions.

Sizes of stomata were analyzed on high resolution (4096 × 3288 pixel) images, where the size scale was measured with the help of a calibration slide. Measurements were performed along with the longitudinal axis of each stoma.

### 4.3. Determination of Stress Enzyme Activity

The peroxidase class III (POD; EC 1.11.1.7) isoform activity, which is sensitive to abiotic stress but also shows large variation depending on genetic background, was determined based on Rao et al. [50] with modifications by Solti et al. [47]. A 900 mg leaf sample was powdered in liquid nitrogen, homogenized with 4 mL ice-cold isolating buffer (50 mM Na-K-phosphate buffer, pH 7.0, 1.0 mM EDTA, 0.1% (*w*/*v*) Triton X-100), and centrifuged at 4 °C with 20,000× *g* for 20 min to pellet cell wall debris, organelle, and other membrane fragments. Supernatant was collected as crude extract. To separate native soluble proteins, aliquots were deionized in 5 mM Tris-HCl, pH 6.8, 0.01% (*m*/*v*) SDS, 10% (*v*/*v*) glycerol and 0.001% (*m*/*v*) bromophenol blue. The POD isoforms were separated on 10–18% gradient polyacrylamide gels containing 0.01% (*w*/*v*) SDS in the cathode buffer according to Solti et al. [47]. POD isoforms were stained in 50 mM acetate buffer, pH 4.5, 2 mM benzidine, and 3 mM H_2_O_2_. After deionization and 30 min staining, enzyme activity was terminated in 50% (*v*/*v*) methanol, and gels were scanned using an Epson Perfection V750 PRO gel scanner. Densitometry and retention factor (R_f_) calculation were performed by Phoretix v 4.0 software.

POD activity was normalized based on the total soluble protein content of the samples. Protein content was determined by comparative densitometry according to Sárvári et al. [51]. Briefly, proteins were solubilized in in 5 mM Tris-HCl, pH 6.8, 0.1% (*m*/*v*) SDS, 2% (*m*/*v*) dithiothreitol, 10% (*v*/*v*) glycerol, and 0.001% (*m*/*v*) bromophenol blue. Proteins were separated on 10–18% gradient polyacrylamide gels. Proteins were stained by colloidal Coocmassie staining, scanned by Epson Perfection V750 PRO, and evaluated in Phoretix v 4.0 (Phoretix International, Newcastle upon Tyne, UK).

### 4.4. Calculation of Air Pollution Tolerance Index

As an indirect response of plants, the levels of air pollution can be described by the air pollution tolerance index (APTI). An increased APTI value indicates low sensitivity, while reduced APTI values can be considered as indicators of biocontamination [52,53,54,55,56]. The following Equation (1) was applied based on Singh et al. [36] studies:

APTI = [A × (T + P) + R]/10(1)

The values of APTI were calculated on the basis of leaf ascorbic acid content (mg g^−1^) (A), total chlorophyll content (mg g^−1^) (T), leaf extract pH (P), and relative water content of the leaves.

The content of ascorbic acid was measured by the redox titration procedure, where 2 g of leaf tissue was ground and homogenized in 3–4 parts of 50 mL of water. Afterwards, the extract was collected and made up to 100 mL in volumetric flasks. Using this extract, the pH of the leaf was first recorded using a digital pH meter. Following pH measurement, 20 mL of the sample was titrated three times with 0.0025 mol iodine in diluted 1 mL 0.5% starch solution. The blue color remained for 20 s. Approximately 50 mg of chlorophyll was extracted from fresh leaves using 5 mL of 96% ethanol. The absorption of the extracts was measured by absorbance at 653, 666, and 750 nm using spectrophotometric analysis. The overall chlorophyll content (T) was calculated using the formula of Equation (2):

T (mg g^−1^) = (17.12 × E666 − 8.68 × E653) × V/m ×1000 (2)

where V is the leaf extract volume (ml), m is the fresh weight of the leaf sample (g), and E666 and E653 are the absorbance values corrected for extinction at 750 nm.

To determine the pH, 2 g leaf tissue was ground and deionized in 100 mL deionized water. The relative water content (R) was calculated as the Equation (3):

R (%) − (FW − DW) − (TW − DW) × 100(3)

For the determination of the relative water content, the fresh weight (FW) of each leaf was measured. Subsequently, the leaves were immersed in water overnight and then measured again to determine the turgid weight (TW). Lastly, the leaves were dried in an oven at 70 °C in order to measure the dry weight (DW).

### 4.5. Analysis of miRNA-Based Markers

The samples of leaves were homogenized in liquid nitrogen. The complete genomic DNA was isolated by commercial isolation kit NucleoSpin Plant II (Macherey-Nagel™, Düren, Germany) according to the manufacturer’s instruction. DNA extracts were quantified by the Implen NanoPhotometer^®^ (eLabNext, Groningen, The Netherlands) and diluted to 70 ng µL^−1^. Touchdown PCR was performed in a PCR mix of 20 µL containing 70 ng μL^–1^ of genomic DNA, 10 pmol dm^–3^ of each primer (Table 2), 2 U of DreamTaq DNA polymerase, 0.8 mmol dm^–3^ dNTPs (Bioline, AgroSciences Ltd., Essex, UK), and 1× DreamTaq Buffer (KCl, (NH_4_)_2_ SO_4_, 20 mmol dm^–3^ MgCl_2_, Thermo Fisher Scientific Inc., Washington, DC, USA). The PCR amplification program used the ‘touchdown’ method as follows: initial denaturation at 94 °C for 5 min; 5 cycles of 30 s at 94 °C, 45 s at 64 °C (with a 1 °C decrease in annealing temperature per cycle), and 60 s at 72 °C; 30 cycles of 30 s at 94 °C, 45 s at 60 °C, and 60 s at 72 °C; and the final extension at 72 °C for 10 min. The PCR products were separated on 3% stacking and 15% separating TBE-Urea polyacrylamide gels at a constant power 90 V, 25 mA for 120 min using 1× TBE Running Buffer (Thermo Fisher Scientific Inc., Washington, DC, USA). For size comparison, a 10 bp DNA guide (Invitrogen, Thermo Fisher Scientific Inc., Washington, DC, USA) was used. Polyacrylamide gels were stained with GelRedTM Nucleic Acid Gel stain followed by visualization in the G-Box Syngene electrophoresis documentation system. Analysis of the gels was performed using GeneTools software—GeneSnap version 7.09.17 to identify unique fragments.

Genomic conservation of miRNA sequences and especially the stem–loop region of precursor molecules of miRNA (pre-miRNA) provided an opportunity to develop a novel type of molecular markers. MicroRNA-based genotyping technique as a novel type of marker system was published in 2013 by Fu et al. [40]. Since then, this system has been applied to genotyping applications of *Setaria italica* and in related grass species [46]. Given the origin of sequences of this type of markers, they can be considered as functional markers at the DNA level [40,46,55]. The attributes of miRNA-based markers [40,46] are as follows: good stability due to a direct PCR-based marker system; improved reproducibility and sequence specificity due to high annealing temperature (more than 60 °C) and the use of “touchdown” PCR approach; relatively high polymorphism because of possible random combinations of primers; putative functionality due to their polymorphism nature and the ability to predict phenotypes controlled by miRNAs; and crossgenera transferability potential because of the conservation level of miRNAs between species and the way of deriving markers from the consensus sequences of miRNAs.

### 4.6. Flow Cytometric Analyses

The ploidy level can also be an important measure to assess the condition of old tree specimens. Different ploidy levels may indicate different levels of stress tolerance [56]. The ploidy level of samples of *Gingko biloba* were quantified by flow cytometric analyses using a CytoFLEX Flow Cytometer (Beckman Coulter Inc., Brea, CA, USA). The experiments were repeated at least three times.

The leaf samples (25 mg per plant) were harvested from young leaves of the “Acsád”, “Budapest”, and “Keszthely” trees, which were also supplemented with controls (haploid, diploid, and triploid). Leaf sample of a seed-derived *Ginkgo biloba* tree was collected for use as a control (2n), while ‘Chris Dwarf’ and ‘Knapek’s triploid’ varieties were applied as haploid (n) and triploid (3n) controls, respectively. The ploidy levels of these varieties (n and 3n) were published by Šmarda et al. [19].

The samples were chopped with razor blade in 1 mL AE buffer (9.53 mM MgSO_4_ × 7H_2_O, 47.67 mM KCl, 4.77 mM HEPES, 6.48 mM dithiothreitol, 0.25% Triton X-100, pH 8.0) for 1 min.

The above prepared suspensions were purified using 20 µm sieves, and 10 µL RNase (1 mg ml^−1^) solution was then added to each sample at 4 °C for 60 min to remove the RNA content. The DNA content was stained with 40 µL PI solution (1 mg ml^−1^) at 4 °C for 30 min, and the samples were then assayed with a flow cytometry. The level of ploidy in the samples after flow cytometric analysis was determined based on histograms.

## 5. Conclusions

*Ginkgo biloba* is a species that has existed for at least 34 million years and is currently experiencing a renaissance: the basic species and its constantly expanding varieties can be found in countless urban green spaces, including roads and in parks and home gardens. It is an excellent city-tolerant plant, as well as a decorative ornamental plant, and an important fruit-bearing plant in Asia. In Europe, including Hungary, one can find many old specimens, which can now be considered heritage trees, and many of them are protected. There are descriptions of how these now Methuselah-age trees were planted in Europe—and these specimens still live and decorate the parks and public spaces. Under the extremely continental climate of the Carpathian Basin, many species are unable to survive due to the summer drought, extreme temperature maxima, and uneven rainfall distribution. The old *Ginkgo biloba* trees, on the other hand, have survived many historical decades and are still decorative today. By examining their resistance to abiotic stress with various physiological, micromorphological, and genetic methods, the current state of the trees was estimated. According to these results, the physiological state of the trees is adequate, and the next measurements were to assess their genetic properties. This was important from several points of view—the genetic relationship between the trees could be examined, which, up to now, there was no insight into either, but also, how the genetic relationships and physiological stress tolerance are related in the case of old specimens of *Ginkgo biloba* was certainly important too. To this day, *Ginkgo biloba* is one of the most stable and popular species in public areas, and it can withstand environmental stress very well, even in its old age. It can be recommended in urban plantings, where it will probably decorate for decades or centuries.

Finding answers to these questions was a noble task. *Ginkgo biloba* is a mysterious species, and this series of measurements also proved that it is still surrounded by many mysteries. These results can contribute to the rich information base of historical trees. It is a never-ending task that is an important part of sustainable green space management—a multidisciplinary knowledge that brings together the fields of horticulture, landscape architecture, biology, and genetics. We need to be prepared for the effects of urbanization and climate change. Hungary’s often extreme continental climate may provide scope for the identification of highly tolerant *Ginkgo biloba* varieties and their role in green space management. The use of healthy, high-tolerance plants promotes the mental and physical health of city dwellers and enriches the urban ecosystem.

## Figures and Tables

**Table 1:** The observed APTI values for the tested specimens.

Specimen	RWC	AAC	pH	Chlorophyll	APTI	Ganguly et al. [35]	Singh et al. [36]
“Acsád”	73.384	4.625	4.28	15.653	17	intermediate	intermediate
“Budapest”	82	2.635	4.517	17.710	14	sensitive	sensitive
“Keszthely”	83.513	3.787	4.70	21.982	18	intermediate	intermediate

**Table 2:** Sequences of miRNA-based markers applied in the genomic analyses [57].

Markers	Sequences
lus miR168_F	CACGCATCGCTTGGTGCAGGT
lus miR168_R	CCAGTGCAGGGTCCGAGGTA
lus miR408_F	GGCTGGGAACAGACAGAGCATGGA
lus miR408_R	GGGAAAAAGGCCAGGGAAGAGG

## Data Availability

Data are contained within the article.
